# Supplementary material for: Empirical evidence on the efficiency of backward contact tracing in COVID-19
Source: Nat Commun. 2022 Aug 13;13:4750. doi: 10.1038/s41467-022-32531-6 (PMC9375086; doi:10.1038/s41467-022-32531-6)
Supplement: Supplementary file 1 — Supplementary Information [file 41467_2022_32531_MOESM1_ESM.pdf]

Empirical evidence on the efficiency of backward contact tracing in COVID-19

Supplementary Figures

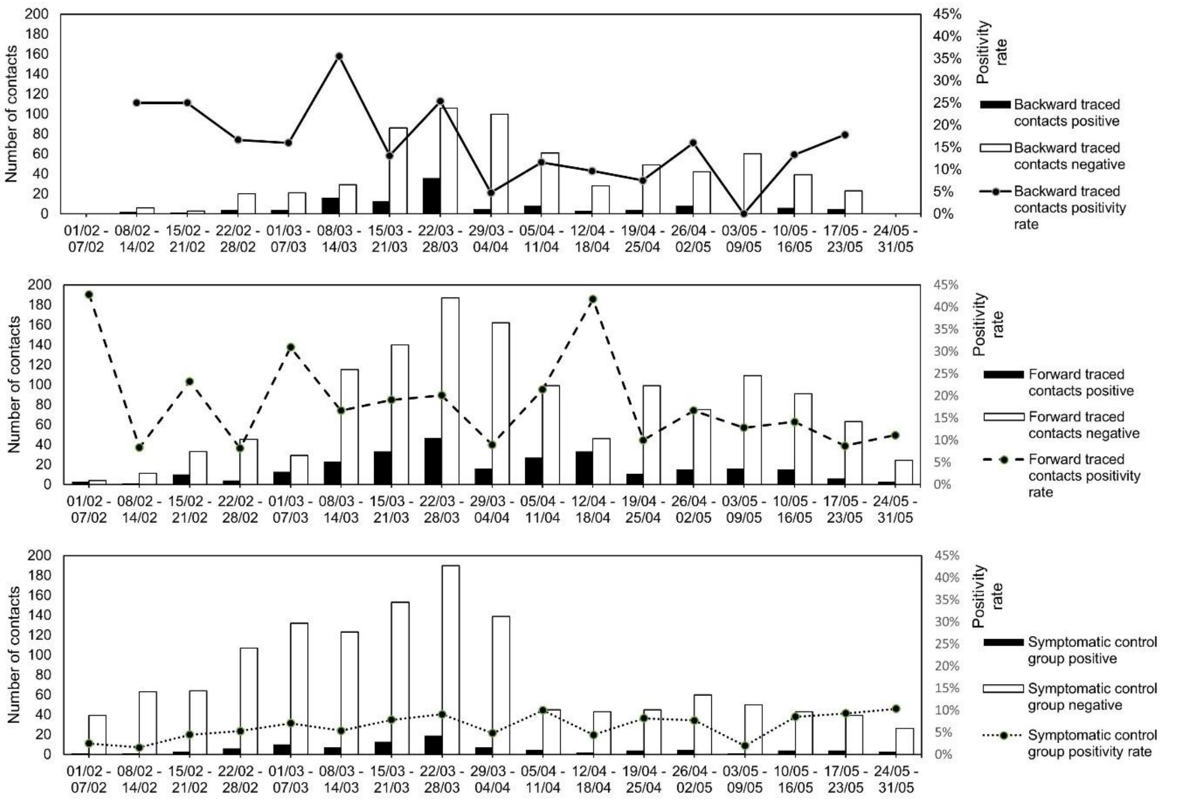

Supplementary Fig. 1. Evolution of control group and contact outcomes over the course of the main study period from February 1st to May 31st 2021. (a) Backward traced contacts. (b) Forward traced contacts. (c) Symptomatic control group.

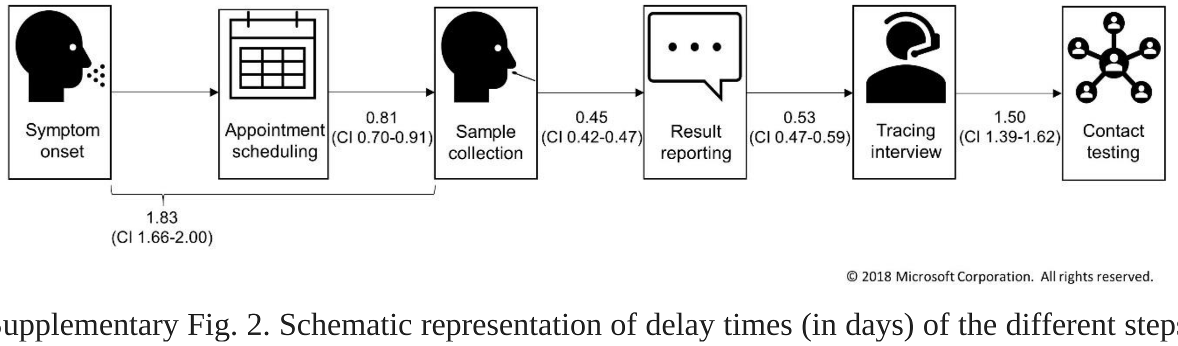

Supplementary Fig. 2. Schematic representation of delay times (in days) of the different steps in the manual test and trace cascade for cases included in the main analysis, with the corresponding confidence intervals (CI). The short delay between symptom onset and sampling

highlights the population's tendency to rapidly self-refer for testing, supported by community engagement and risk communication[1]. The delay was shorter than it was nationally[2]. Only instances in which symptom onset preceded sampling were taken into account. The short delay between appointment scheduling and sample collection reflects highly accessible testing. Only symptomatic individuals were taken into account since they required a test as soon as possible after symptom development. The short delay between sample collection and result reporting results from frequent transport of samples from the testing centre to the lab, rapid turn-around-times and automated result reporting. The short delay between result reporting and both the tracing interview and the first testing of a contact result from the recruitment of a flexible workforce, the introduction of extended timetable for tracing (9AM - 9PM daily) and the active support of self-tracing and digital contact tracing through community engagement and risk-communication. Only contacts who were tested in the university test centre were included when computing the latter delay period.

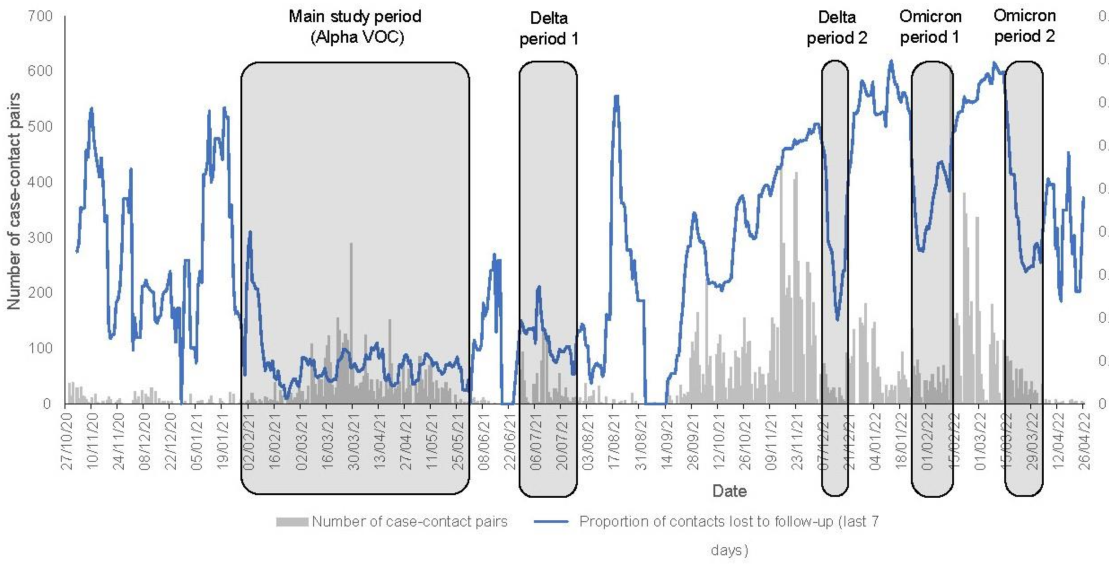

Supplementary Fig. 3. Evolution of the number of case-contact pairs and lost to follow-up rate in the study population. The lost to follow-up rate was used as a marker to select periods of interest for analysing the impact of changes in dominant circulating variant of concern (VOC). Grey columns show the number of case-contact pairs identified daily by the university contact tracing team. The trajectory shows a run-in period during the second wave of COVID-19 in the fall of 2020, during which there was likely significant under-detection of cases and contacts[3]. The third (Alpha VOC), fourth (Delta VOC) and fifth (Omicron VOC) waves can be seen in spring 2021, fall 2021 and winter 2021-2022, respectively. The proportion of contacts lost to follow-up is shown in blue. In addition to the initial analysis focusing on the Alpha dominant period (from February 1st until May 31st 2021), periods of interest were selected during the clear dominance of a particular VOC (Supplementary Figure 10) if they were characterised by a low lost to follow-up rate. Amongst other factors, the epidemiological trajectory, the availability of contact tracing manpower and government-mandated testing policies determined whether contacts were systematically followed up and their outcomes recorded.

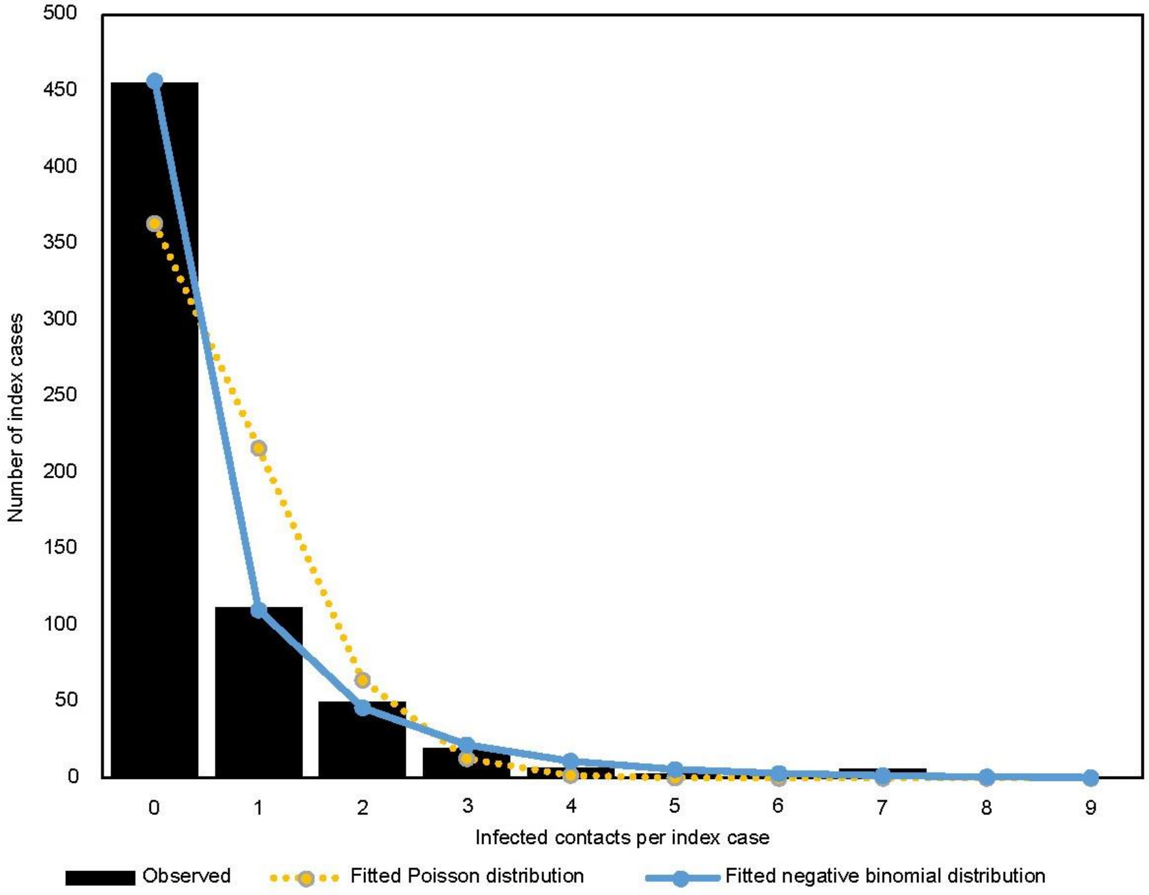

Supplementary Fig. 4 shows the distribution of the number of infected contacts per index case, using only the included case-contact pairs in the main study period. This is different from the offspring distribution, as no assumption is made on the relative position of the index case and their contact in the transmission tree. A Poisson distribution (reflecting homogenous distribution of infected contacts, yellow on the graph) does not fit our data well. The data does fit with a negative binomial distribution (blue on the graph) with mean 0.59 (average infected contacts per case, variance 1.5) and dispersion parameter k=0.40 (standard error 0.06).

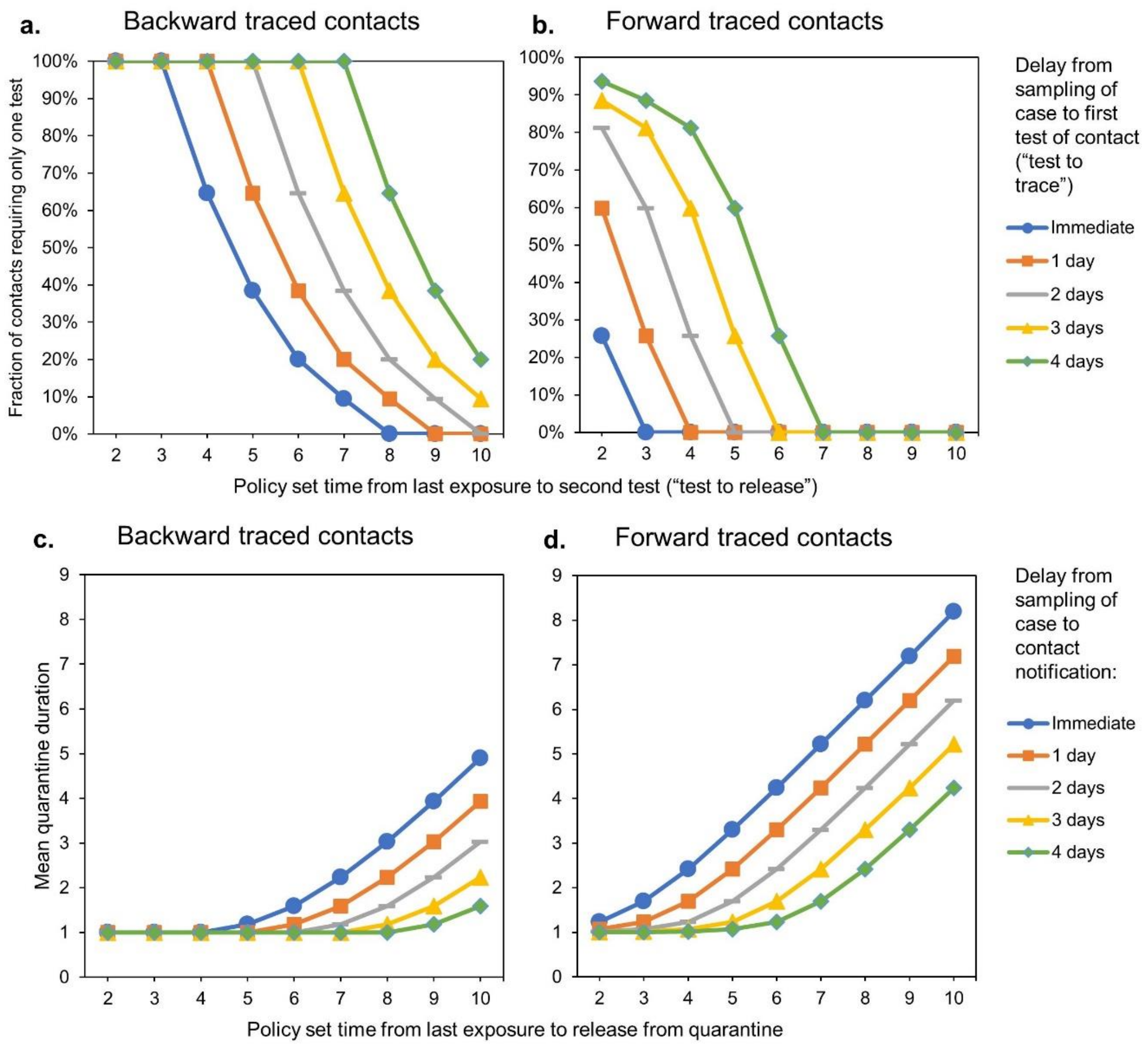

Supplementary Fig. 5. Simulation of the number of tests and quarantine days required for identified contacts of a case, based on last exposure dates of contacts in the main study period. Panel a and b show the fraction of backward and forward contacts respectively, who only require a single test for both "test to trace" and "test to release" purposes. The policy set minimal delay required between last exposure and "test to release" is shown on the *x*-axis. This delay is ideally chosen based on sensitivity of the diagnostic test and the accepted risk of post-quarantine transmission. Different colours and markers show a range of combined testing and tracing delays, varying from immediate, which in practice may only be possible with point of care tests, to 4 days. As the delay from testing of an index case to the first test of their contacts increases and as the policy set minimum delay between last exposure and a valid "test to release" is shortened, a higher proportion of individuals can leave quarantine after a single combined "test to trace" and "test to release". Given identical delays and policy, this percentage is much higher in backward traced contacts than forward traced contacts, due to inherent differences in exposure dates. As a result, the testing burden in backward as opposed to forward traced contacts is lower. Panel c and d show the mean quarantine duration for backward and forward traced contacts respectively, assuming a minimal duration of 1 day to allow for contact testing. Given the dates of last exposure from our dataset, quarantine duration depends on tracing delays and the policy set time from last exposure to release from quarantine. For most

combinations of these variables, mean quarantine duration is considerably lower for backward as opposed to forward traced contacts.

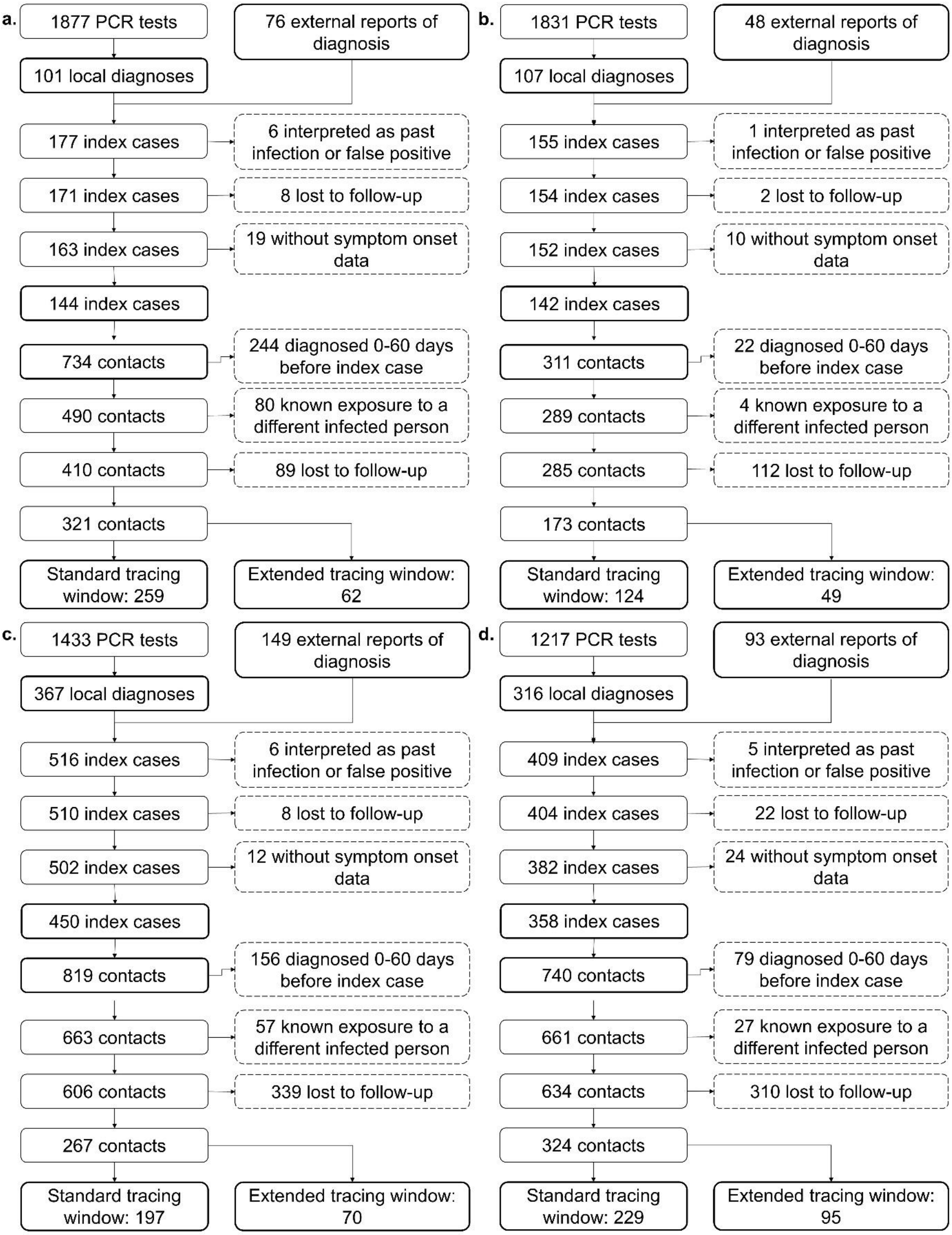

Supplementary Fig. 6. Exclusion flow charts for the number of cases and contacts included and excluded in the four periods as highlighted in Supplementary Figure 3. Panels (a), (b), (c) and (d) show the exclusion flowcharts for individuals included during the first Delta dominant,

second Delta dominant, Omicron BA.1 dominant and Omicron BA.2 dominant periods, respectively.

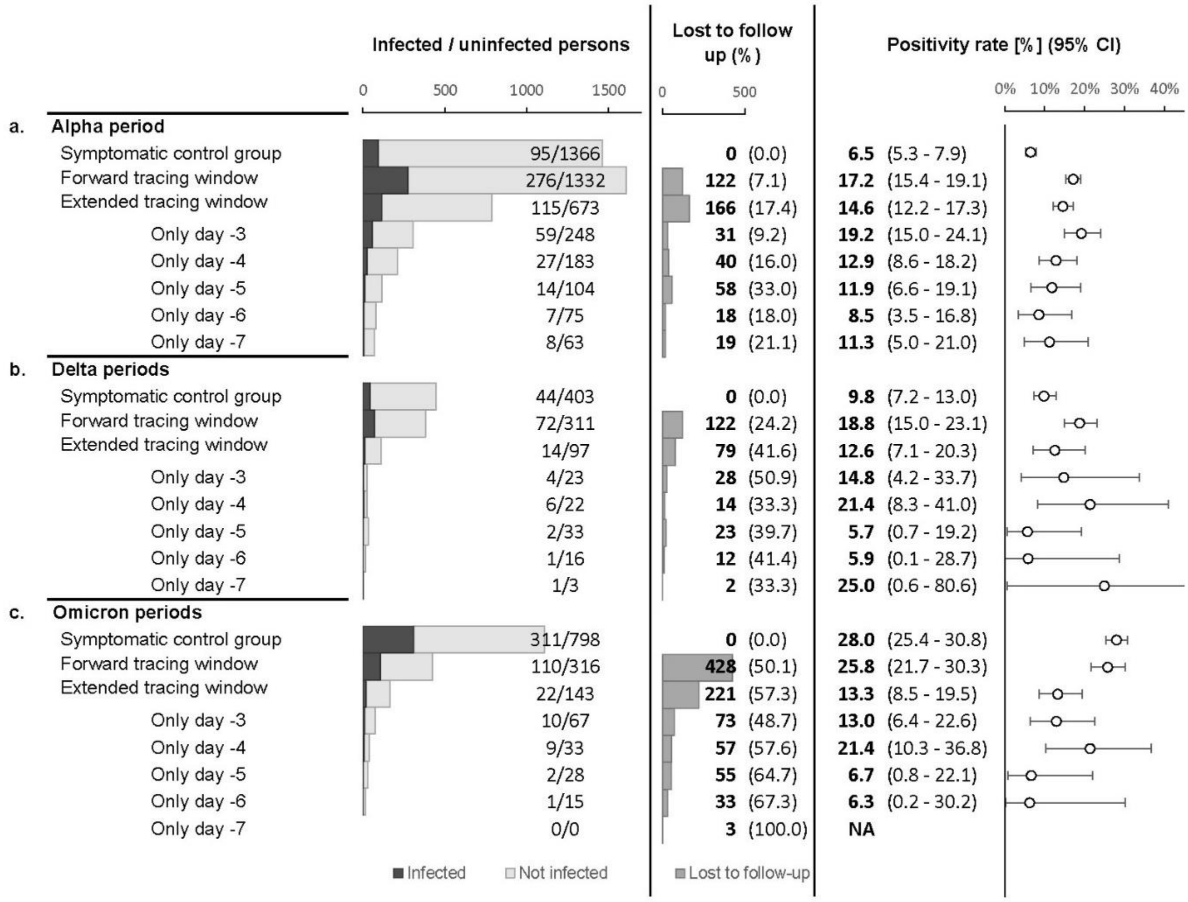

Supplementary Fig. 7. Outcomes and positivity rates for contacts in the main study period and subsequent periods of interest, each sub-grouped by the day of last exposure to the index case, relative to the index case's onset or sampling date. Panel (a) repeats the main study outcomes from Figure 4 panels (b) and (c). Panel (b) shows results for the periods with high follow up rates during which the Delta variant was dominant. Panel (c) shows results for the periods with high follow up rates during which the Omicron variant was dominant. Case-contact pairs were included by means of equivalent inclusion and exclusion criteria as during the Alpha period (Figure 3 and Supplementary Figure 6). In addition to lower case numbers, the interpretation of risks in more recent study periods is hindered by higher lost to follow-up rates and reduced reliability of the symptomatic control group. Both may have resulted from the loosening of government-mandated testing criteria, the reduced tendency of contacts to be tested after vaccination and the rollout of pharmacy-based and self-administered rapid antigen tests, the results of which were not directly captured by our system[1,2,7,8].

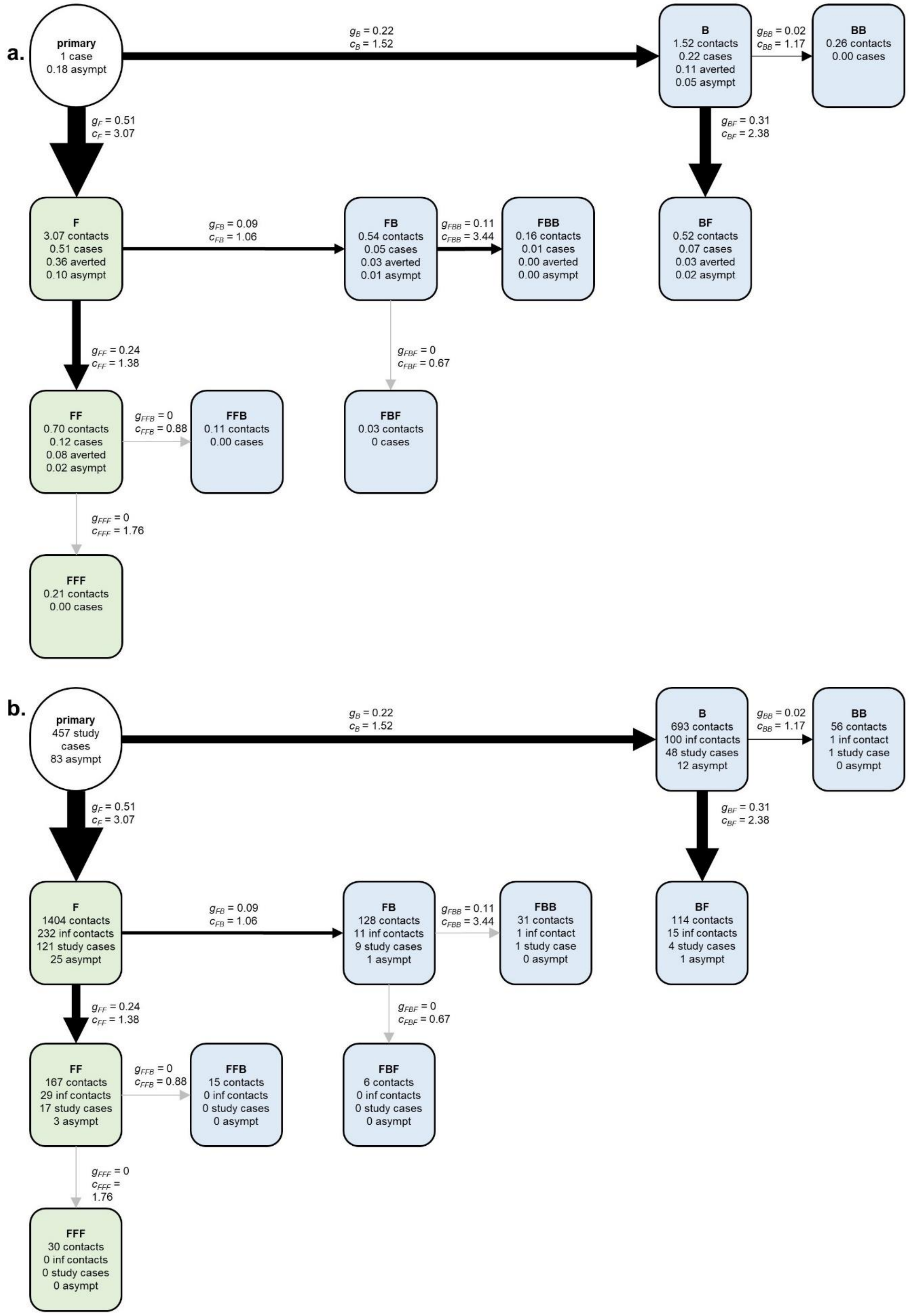

Supplementary Fig. 8. Modelled tracing sequences in a branching process model of iterative contact tracing. The number of contacts, infected contacts, study cases and asymptomatic study

cases is shown according to the tracing sequence (box) which identifies each. For example, 'FB' indicates backward traced contacts of infected forward traced contacts of a primary index case. Sideways and downward arrows indicate backward and forward traced contacts from the previous generation respectively. Green: groups identified through forward iterative contact tracing. Blue: groups identified only through backward iterative contact tracing. Panel a) shows the observed numbers during the main study period. Study cases are infected contacts who were contacted by our tracing team because they were part of the target population. Primary study cases are study cases who were not previously identified as a contact of a student in the target population. Asympt indicates study cases who were asymptomatic at the time of their tracing interview. Each arrow is labelled with the observed number of cases g_X and number of contacts c_X detected per index case of the previous tracing step. Both are assumed to be zero if the number of study cases in the previous generation is lower than or equal to 5. Arrow thicknesses correspond to g_X. Panel b) shows the number of contacts and cases predicted by the model. The obtained values of c_X and g_X are used as parameters in the model, to determine the expected numbers of contacts, cases, asymptomatic cases and averted infectiousness per primary index case in each tracing sequence, had all reported contacts been traced as the study population was.

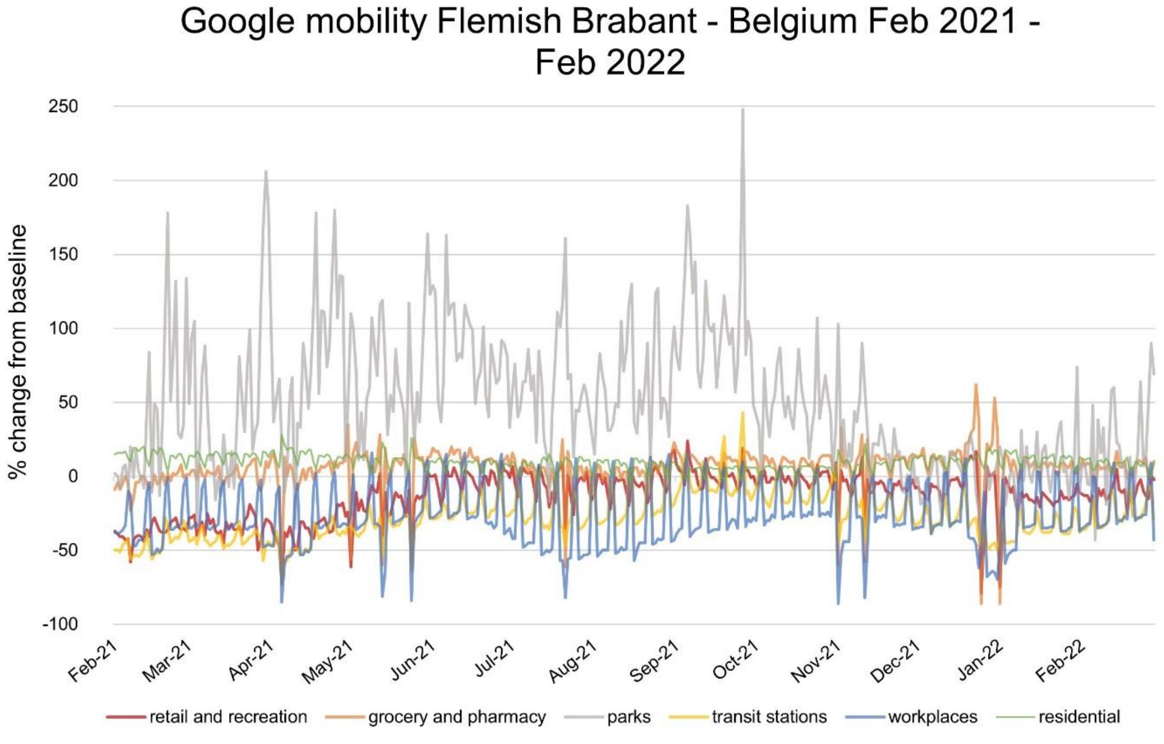

Supplementary Fig. 9. Google mobility data between February 1st 2021 and February 31st 2022 for the region of Flemish Brabant, Belgium. Changes in mobility patterns are expressed as percentage changes in number of visits to particular places of interest, grouped by category, in the region relative to baseline levels at the start of 2020. For the 'residential' category, the % change in total time spent as opposed to baseline is shown. They demonstrate the progressive normalisation of mobility patterns over the course of 2021.[10]

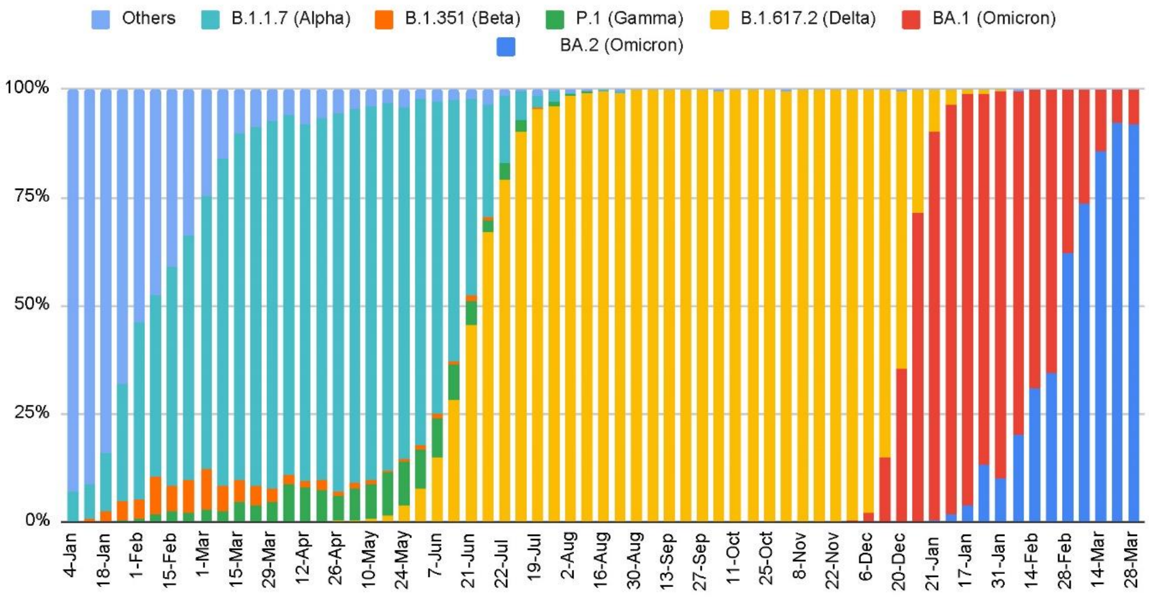

Supplementary Fig. 10 shows the distribution of variants of concern in Belgium from January 2021 until March 2022[13].

Supplementary Tables

	Benefits			Costs		
Strategy	Cases detected	Asympt cases detected	Infectivity averted	Contacts traced	Contact tests	Quarantine days
Forward tracing	1	1	1	1	1	1
Backward tracing	0.55	0.61	0.38	0.78	0.67	0.40
Day -3	0.31	0.36	0.23	0.32	0.32	0.23
Day -4	0.11	0.04	0.07	0.18	0.17	0.09
Day -5	0.06	0.11	0.04	0.15	0.12	0.05
Day -6	0.03	0.04	0.02	0.07	0.03	0.01
Day -7	0.03	0.06	0.02	0.07	0.04	0.02

Supplementary Table 1. Benefits and costs of backward iterative tracing in a simple branching process model (described in Supplementary Methods). Asympt: asymptomatic. Benefits gained are shown as additional cases detected, asymptomatic cases detected and infectivity averted, while costs incurred are quantified as additional contacts traced, contact tests and quarantine days per day added to the backward contact tracing window. The benefits and costs of forward iterative contact tracing are taken as reference. A distribution of timing of transmission relative to symptom onset is used to determine the averted infectivity[9].

Supplementary Methods

Target population size estimate

The target population consisted of higher education students living or studying in the city of Leuven, the eight largest city in Belgium at around 102,000 inhabitants. Leuven is located in the province of Flemish Brabant in Belgium, 25 km east of Brussels. Its higher education institutions accommodated 56,390 students on their Leuven campuses in the main study period. The KU Leuven association, the largest institution, had 56,099 students enrolled in its Leuven based campuses at the start of February 2021. Other institutions Vlerick Business School and the Evangelical Theological Faculty had 63 and 228 enrolments respectively.

40,144 students attached to the KU Leuven association had an official address or a student room in the city of Leuven or its surrounding communes. Extrapolated for the other higher education students in the Leuven region, for which we did not have residency addresses available, a total of 40,352 tertiary education students were estimated to have an address in the Leuven area.

Generalised contact restrictions and the limitation of in-person teaching in the main study period likely prompted some students to reside outside of the Leuven area during the second semester of the 2020-2021 academic year, reducing the actual size of the target population. To estimate the true fraction of students present, we looked at our contact tracing information. Any positive case triggered the investigation of students sharing common living areas with the index case. From this data, we calculated that 76.1% of students were physically present at their student room during the study period. When taking this into account, the average target population was estimated at 32,965 students.

General contact restrictions in study period

Existing general contact restrictions have a major impact on any contact tracing program's effectiveness and efficiency by influencing the type and number of contacts an index case encounters prior to and during their infection.

One measure for assessing general contact restrictions are the Google community mobility reports[10]. In the province of Flemish Brabant, where the city of Leuven represents almost 9% of the population, the frequency of visits during the main study period as a whole was reduced by 31% for retail and recreation, 41% for transit stations and 30% for workplaces in comparison to baseline levels. Visits were increased by 2% for grocery and pharmacy and 59% for parks in comparison to baseline levels. Time spent in residential buildings was increased by 13% during the study period. During later periods, generalized contact restrictions were generally less stringent than they were in the main study period, as can be seen in Supplementary Figure 9 below.

Containment and closure policies can also be summarised by the Oxford COVID-19 Government Response Tracker (OxCGRT) stringency index[11]. This is an aggregate score quantifying containment and closure policies, sometimes referred to as lockdown policies. The stringency index varied from 62.96 at the start of the main study period to 75.93 during a period of strengthened measures around Easter and 54.63 from the start of May onwards. The remainder of 2021 and start of 2022 saw a general reduction of the stringency index[12].

For tertiary education students specifically, occupancy levels of in-person educational activities were limited to 10% of the usual levels from February until halfway April and increased to 20% thereafter.

Other public health programs focussing on testing & contact tracing

Our program ran in parallel with existing contact tracing programs at the level of the Flemish region and the city of Leuven. During the main study period, the Flemish program focused on contact tracing with testing both immediately and 7 days after exposure for contacts last encountered by the index case up to two days prior to symptom onset or diagnosis[2]. The city of Leuven focused on the containment of clusters of infections in congregate care settings and schools. Outbreaks in student residences were investigated by the KU Leuven contact tracing program.

Variants of SARS-CoV-2 in study cases

Throughout the main study period (February until May 2021), the B.1.1.7 (Alpha) variant was dominant on a national level. This changed in the study periods thereafter (Supplementary Figure 10).

Presence of immunity in the population under study

Test centre data show that the percentage of students reporting having received at least one COVID-19 vaccine increased from 2.8% (18 out of 611 and 118 out of 4211 respectively) in February and March to 8.6% (250 out of 2913) in April and 10.2% (182 out of 1776) in May 2021. These numbers are in line with the data publicly available for the Flemish region, showing that by the start of February, 2.47% of the 18 to 34 year old Flemish had received at least one dose of a COVID-19 vaccine, which increased to 5.46% in the beginning of March, 11.1% in the beginning of April, 13.4% in the beginning of May and 17.1% in the beginning of June[14]. From September onwards, the percentage of students reporting having been fully vaccinated reached 90%. The rollout of booster doses was initiated at the end of 2021. Test centre data show that the percentage of students reporting having received a booster dose of a COVID-19 vaccine increased to 29% in January 2022 and 54% in February 2022 (1010 out of 3500 and 2411 out of 4500 respectively).

Description of iterative contact tracing model

Many of the reported contacts in our dataset were outside the study population, which means their infected contacts were not iteratively traced using the same backward tracing strategy.
To estimate how efficient backward contact tracing would be if all infected contacts were iteratively traced, we used a simple deterministic branching process model. Instead of modelling the entire transmission tree, we limited the branching process to traced cases and contacts, where each subsequent generation consisted of the contacts traced from the previous generation. This allowed us to avoid making assumptions on the direction of transmission or the probability of an infected contact being traced.

Using the observed numbers of contacts and infected contacts per case detected according to each iterative tracing sequence, the model extrapolated our observations to estimate the total number of traced contacts in a hypothetical situation where all infected contacts were traced using an extended tracing window strategy. The model assumed that tracing someone has perfect effectiveness in preventing transmission from the moment of tracing onwards, i.e. transmission after tracing is impossible. The same exclusion criteria applied as in the rest of the study. For example, a contact of multiple cases was only included as a contact of the first case to report them.

We investigated the effect of two strategies: forward tracing only and an extended tracing window. The benefit outcomes of the model were the expected number of detected contacts, detected asymptomatic contacts and averted transmission potential per primary index case. The cost outcomes were quantified as the expected number of traced contacts, quarantine days and contact tests.

The model started from a single primary index case, which is an index case who was not previously identified as a contact of a student in the target population. Each subsequent tracing generation split into two branches representing the number of forward and backward traced contacts from the previous generation.

We grouped traced contacts according to the contact tracing sequence which identified them. The group with sequence $X = (a_1, \dots, a_n)$ consisted of contacts identified through n tracing generations, where each item a in the sequence had a value of either F or B, indicating a forward or backward tracing step from the previous generation respectively. For example, a backward traced contact of an infected forward traced contact of the primary index case was assigned to the group with sequence (F, B).

$G(X)$ is the number of infected contacts identified in the last step of the tracing sequence X and its expected value was determined as follows:

$$\begin{aligned} G(X) &= G(X_{\hat{n}}) \times g_X \\ &= \prod_{i=1}^{n} g_{(a_1,\dots,a_i)} \end{aligned} \tag{1}$$

where $X_{\hat{n}} = (a_1, \dots, a_{n-1})$ is tracing sequence X without the last step, while g_X is the number of infected contacts identified via the last tracing step a_n per case of the previous generation $X_{\hat{n}}$. Similarly, the total number of contacts detected in the last step of X is given by:

$$C(X) = G(X_{\hat{n}}) \times c_X \tag{2}$$

with c_X the number of traced contacts per case of the previous generation $X_{\hat{n}}$, i.e. in the step a_n.

The total number of detected infections, not including the primary index case, across all tracing generations, is given by G_{tot}.

$$G_{tot} = \sum_j G_j \tag{3}$$

We estimated c_X and g_x from our data, by determining the number of observed forward and backward traced contacts and cases per study case traced according to $X_{\hat{n}}$ (Supplementary figure 8). If the number of study cases traced according to $X_{\hat{n}}$ was smaller than or equal to 5,

c_X and g_X were assumed to equal 0. This was done to avoid the model becoming too sensitive to outliers when the number of observations to determine g_X was small.
We estimated the fraction of averted transmissions $a(x)$ from each detected case x as follows:

$$\begin{aligned} a(x) &= Pr\left(T > t_{test_p} + d_{trace}\right) \\ &= 1 - F_T\left(t_{test_p} + d_{trace}\right) \end{aligned} \tag{4}$$

Here, T is the timing of onward transmission relative to symptom onset of the detected case, $F_T(t)$ is its cumulative distribution function, t_{test_p} is the timing of parent case testing relative to symptom onset of the detected case and d_{trace} is the delay from parent case sampling to contact notification, assumed 0. We determined $F_T(t)$ using a distribution of transmission timing[9]. Asymptomatic infected contacts were assumed to be detected at the same time in their infectiousness cycle as symptomatic infected contacts. The number of quarantine days $q(x)$ for each contact was calculated by assuming that contacts were instructed to quarantine from the time of contact notification $\left(t_{test_p} + d_{trace}\right)$ until a delay $d_{quarantine}$ after the last reported exposure to the index case t_{exp} or until a first test delay d_{test_1} (1 day) after notification, whichever was later. (5)

$$q(x) = \max\left(\left(t_{exp} + d_{quarantine}\right) - \left(t_{test_p} + d_{trace}\right),\ d_{test_1}\right)$$

The number of required tests $s(x)$ per contact, in case of a combined “test to trace” and “test to release” strategy, was calculated by assuming that a single test suffices when the timing of the first test is after or on the same day as the second test:

$$s(x) = \begin{cases} 1, & t_{test_p} + d_{trace} + d_{test_1} \geq t_{exp} + d_{test_2} \\ 2, & otherwise \end{cases} \tag{6}$$

d_{test_2} is the policy set time from last exposure to the second test, which releases the contact from quarantine if negative.
The fraction of asymptomatic infected contacts $f_{asympt}(X)$ was also determined from our study case data.

The pseudocode below represents the required computations in a stepwise fashion:

```
Set the tracing window under consideration, in days relative to symptom onset
or test (whichever was earlier) of an index case.

For every study contact i in the main study period:
  Assign i to a group X according to their tracing sequence  Exclude i if
their tracing sequence involved any step outside of the tracing window under
consideration
  Calculate i's number of quarantine days q_i = time from detection to release
  Calculate i's number of tests s_i = if test2_date > test1_date: 2 tests,
else 1 test
  If i is infected:
    Calculate i's fraction of averted transmissions a_i from the distribution
of transmission timing

For every group X of contacts with a certain tracing sequence:
```

```
  Calculate q_X = mean of q_i
  Calculate s_X = mean of s_i
  Calculate a_X = mean of a_i (infected contacts only)
  Calculate n_study_contacts_X = the total number of contacts in the group
  Calculate n_inf_study_contacts_X = the total number of infected contacts
in the group
  Calculate n_study_cases_X = the total number of infected contacts in the
group, who were also part of the study population
  Calculate f_asymp_X = the fraction of asymptomatic cases amongst the
contacts who were study cases

For every group X, calculate the observed number of contacts and infected
contacts per study case in the previous generation:
  If n_study_cases in X-hat >= 5:
    Calculate c_X = n_study_contacts_X / n_study_cases_X-hat
    Calculate g_X = n_inf_study_contacts_X / n_study_cases_X-hat
  Else:
    c_X and g_X both equal 0

For every group X, use the values of c_X and g_X to calculate the expected
number of cases and contacts in the group:
  Exp_cases_X = g_X * Exp_cases_X-hat
  Exp_contacts_X = c_X * Exp_cases_X-hat

For every group X, use the expected number of cases and contacts to calculate
overall results for the group:
  Exp_q_X = Exp_contacts_X * q_X
  Exp_s_X = Exp_contacts_X * s_X
  Exp_a_X = Exp_cases_X * a_X
  Exp_asymp_cases_X = Exp_cases_X * f_asymp_X

Sum the totals across all groups X:
  Exp_contacts = sum of all Exp_contacts_X
  Exp_cases = sum of all Exp_cases_X
  Exp_q = sum of all Exp_q_X
  Exp_s = sum of all Exp_s_X
  Exp_a = sum of all Exp_a_X
  Exp_asymp_cases = sum of all Exp_asymp_cases_X
```

References

1. Raymenants, J. *et al.* Integrated PCR testing and extended window contact tracing system for COVID-19 to improve comprehensiveness and speed.
2. Proesmans, K. *et al.* COVID-19 contact tracing in Belgium: main indicators and performance, January – September 2021. *Arch. Public Heal. 2022 801* **80**, 1–10 (2022).
3. COVID-19 Data Explorer - Our World in Data. https://ourworldindata.org/explorers/coronavirus-data-explorer?zoomToSelection=true&time=2020-03-01..latest&facet=none&pickerSort=asc&pickerMetric=location&Metric=Confirmed+cases&Interval=7-

day+rolling+average&Relative+to+Population=true&Color+by+test+positivity=false&country=USA~GBR~CAN~DEU~ITA~IND.
4. R, L. *et al.* Epidemiology and transmission dynamics of COVID-19 in two Indian states. *Science* **370**, (2020).
5. Adam, D. C. *et al.* Clustering and superspreading potential of SARS-CoV-2 infections in Hong Kong. *Nat. Med.* **26**, (2020).
6. Bi, Q. *et al.* Epidemiology and transmission of COVID-19 in 391 cases and 1286 of their close contacts in Shenzhen, China: a retrospective cohort study. *Lancet. Infect. Dis.* **20**, 911–919 (2020).
7. Procedures - meest recente wijzigingen | Coronavirus Covid-19. https://covid-19.sciensano.be/nl/procedures/home.
8. Centre for Disease Prevention, E. Evaluation of the SARS-CoV-2 testing policy in Belgium.
9. Ferretti, L. *et al.* The timing of COVID-19 transmission. *medRxiv* 2020.09.04.20188516 (2020) doi:10.1101/2020.09.04.20188516.
10. COVID-19 Communitiy Mobility Reports. *Google* https://www.google.com/covid19/mobility/ (2021).
11. Hale, T. *et al.* A global panel database of pandemic policies (Oxford COVID-19 Government Response Tracker). *Nat. Hum. Behav.* **5**, (2021).
12. OxCGRT. https://covidtracker.bsg.ox.ac.uk/stringency-scatter.
13. Nevejan, L. *et al.* Genomic surveillance report - update for Belgium, April 5th 2022.
14. Dashboard Covid Vaccinations Belgium. *Sciensano* https://covid-vaccinatie.be/en (2021).
