# Supplementary material for: Empirical evidence on the efficiency of backward contact tracing in COVID-19
Source: Nat Commun. 2022 Aug 13;13:4750. doi: 10.1038/s41467-022-32531-6 (PMC9375086; doi:10.1038/s41467-022-32531-6)
Supplement: Supplementary file 3 — Description of Additional Supplementary Files [file 41467_2022_32531_MOESM3_ESM.pdf]

Description of Additional Supplementary Files

File Name: Supplementary Data 1
Description: Code for the iterative contact tracing model described in Supplementary Methods and visualised in Figure 8, Supplementary Fig. 8 and Supplementary Table 1.
