# Supplementary material for: Empirical evidence on the efficiency of backward contact tracing in COVID-19
Source: Nat Commun. 2022 Aug 13;13:4750. doi: 10.1038/s41467-022-32531-6 (PMC9375086; doi:10.1038/s41467-022-32531-6)
Supplement: Supplementary file 5 — Reporting Summary [file 41467_2022_32531_MOESM5_ESM.pdf]

nature portfolio

Corresponding author(s): Joren Raymenants

Last updated by author(s): Aug 2, 2022

Reporting Summary

Nature Portfolio wishes to improve the reproducibility of the work that we publish. This form provides structure for consistency and transparency in reporting. For further information on Nature Portfolio policies, see our Editorial Policies and the Editorial Policy Checklist.

Statistics

For all statistical analyses, confirm that the following items are present in the figure legend, table legend, main text, or Methods section.

n/a	Confirmed	
☐	☒	The exact sample size (*n*) for each experimental group/condition, given as a discrete number and unit of measurement
☐	☒	A statement on whether measurements were taken from distinct samples or whether the same sample was measured repeatedly
☐	☒	The statistical test(s) used AND whether they are one- or two-sided<br>*Only common tests should be described solely by name; describe more complex techniques in the Methods section.*
☐	☒	A description of all covariates tested
☐	☒	A description of any assumptions or corrections, such as tests of normality and adjustment for multiple comparisons
☐	☒	A full description of the statistical parameters including central tendency (e.g. means) or other basic estimates (e.g. regression coefficient) AND variation (e.g. standard deviation) or associated estimates of uncertainty (e.g. confidence intervals)
☐	☒	For null hypothesis testing, the test statistic (e.g. *F*, *t*, *r*) with confidence intervals, effect sizes, degrees of freedom and *P* value noted<br>*Give P values as exact values whenever suitable.*
☒	☐	For Bayesian analysis, information on the choice of priors and Markov chain Monte Carlo settings
☒	☐	For hierarchical and complex designs, identification of the appropriate level for tests and full reporting of outcomes
☒	☐	Estimates of effect sizes (e.g. Cohen's *d*, Pearson's *r*), indicating how they were calculated

Our web collection on statistics for biologists contains articles on many of the points above.

Software and code

Policy information about availability of computer code

Data collection	For data collection we used Go.Data, a contact tracing tool developed by the World Health Organisation. Go.Data version: 2 .37.0 - build 21 0511152 8. Go.Data was integrated with custom appointnemt management, contact listing, and laboratory result modules.
Data analysis	Data analysis was performed either using R script in R version 4.0 .3 or python script in python version 3.8 .specifically written for this study. The code of the iterative contact tracing model is available as a supplementary data file.

For manuscripts utilizing custom algorithms or software that are central to the research but not yet described in published literature, software must be made available to editors and reviewers. We strongly encourage code deposition in a community repository (e.g. GitHub). See the Nature Portfolio guidelines for submitting code & software for further information.

Data

Policy information about availability of data

All manuscripts must include a data availability statement. This statement should provide the following information, where applicable:

- Accession codes, unique identifiers, or web links for publicly available datasets
- A description of any restrictions on data availability
- For clinical datasets or third party data, please ensure that the statement adheres to our policy

The data underlying the main analyses in this manuscript are available in the article and in its online supplementary material. The data that is not released with the paper, and which may require EC approval before sharing, can be made available on request from the corresponding author (Joren

Raymenants), who will respond within four weeks. There must be a demonstrable affiliation with an academic or health institution, a legitimate epidemiological question and a commitment to not attempt to de-anonymise.

Human research participants

Policy information about studies involving human research participants and Sex and Gender in Research.

Reporting on sex and gender	Index cases were 51.1% male (missing data 12.1%).<br>We did not collect demographic data on contacts of index cases.<br>There was a slightly higher percentage of women in the control group (56.6%, missing data 3.0%) compared to the index cases.
Population characteristics	Index cases had a mean age of 21.4 years (SD: 3.60 years, missing data 15.0%).<br>We did not collect demographic data on index cases.<br>the mean age was similar in the control group (22.0 years; SD 3.84 years, missing data 3.0%) as in the index group.
Recruitment	14,917 students underwent RT-qPCR testing at our centre in this period (3.8 tests per 1,000 persons daily), resulting in 498 students with a new diagnosis of COVID-19. A further 231 positive RT-qPCR test results of students in the study population were reported to us from external sources, resulting in a total of 729 cases. 36 (4.9%) of these were interpreted as a past infection or false positive by the treating physician, leaving 693 actual cases (14-day incidence of 245 per 100,000). Six cases (0.9%) were considered lost to follow-up, because they could never be contacted by the contact tracing team, and 28 (4.1%) were excluded because data on presence of symptoms was missing. Therefore, 659 index cases remained in the analysis (Figure 3).<br>Contact tracing of the index cases resulted in 3,971 case-contact pairs (mean 6.0 contacts per case, 2.2 times the national average37), of which 956 (24.1%) were excluded because the contact person already had a positive test result 0 to 60 days before the positive test of the index case. Another 331 (11.0%) contacts were excluded because they already had a known exposure to a different infected individual within 7 days before the tracing interview. Finally, 288 contacts (10.7%) were lost to follow-up.
Ethics oversight	The study protocol was approved by the Ethics Committee Research UZ / KU Leuven. Informed consent was waived as the data gathered did not exceed what was required for the purpose of safeguarding public health

Note that full information on the approval of the study protocol must also be provided in the manuscript.

Field-specific reporting

Please select the one below that is the best fit for your research. If you are not sure, read the appropriate sections before making your selection.

☒ Life sciences ☐ Behavioural & social sciences ☐ Ecological, evolutionary & environmental sciences

For a reference copy of the document with all sections, see nature.com/documents/nr-reporting-summary-flat.pdf

Life sciences study design

All studies must disclose on these points even when the disclosure is negative.

Sample size	The data feeding into this study were gathered in the light of the ongoing public health response for COVID-19. The exact main study period was chosen from February onwards since gradual improvements in data gathering - through updates of the IT infrastructure and human capacity buyilding - allowed for follow-ups of all contacts to be consistently recorded from February onwards. The end of the main study period marks the end of the academic semester, at which point testing and case numbers fell precipitously. The resulting number of cases and contacts is a consequence of the epidemiological trajectory within the study period.<br>For the analyses of more recent cohorts, time periods were chosen according to the main circulating VOC and the lost wo follow up rates of contacts (Supplementary Fig. 3).
Data exclusions	Missing demographic data was ignored in the calculations and the amount of missing data reported. Contacts with missing outcome data were considered lost to follow-up.<br>Cases with missing data on presence of symptoms or symptom onset were excluded, because it was not possible to determine whether they should be included in the extended tracing window group. Cases who could not be contacted by the team were also excluded, as well as cases who were interpreted by their treating physician as not actively infected with SARS-CoV-2.<br>Contacts were excluded if, they had already had a positive test Oto 60 days before the positive test of the index case, assuming that these contacts were either naturally immune or already detected by the healthcare system. Individuals who were already identified as contacts exposed to a previously diagnosed index case within 7 days before the contact tracing interview were excluded as contacts from the second identified index case, while still being considered as contacts for the first.<br>Cases and contacts lost to follow-up were not included in the analysis.<br>In the process branching model, the same inclusion-exclusion criteria were followed. If the number of study cases traced according to tracing sequence X_n was smaller than or equal to 5, c_X and g_X were assumed to equal 0. This was done to avoid the model becoming too sensitive to outliers when the number of observations to determine g_X was small
Replication	Unfortunately, we were unable to replicate the high follow-up rates of the main study period in subsequent periods with different dominant

Replication

variants of concern. We attribute this mainly to gradual loosening of government-mandated testing protocols and higher viral circulation, forcing the contact tracing team to prioritise contact notification over follow-up 45,46 (Supplementary Fig. 3). The control group probably also suffered a further reduction in reliability after the main study period, due to the rollout of alternative testing methods such as pharmacy-based and self-administered rapid antigen tests and the progressive scaling back of RT-qPCR testing in general 37,45,46. Based on follow-up rates, we chose four subsequent periods of interest, characterised by Delta and Omicron VOC dominance, for analysis (Supplementary Fig. 3 and 6). These periods also differed from the main study period with regards to several other factors, such as general contact restrictions, population immunity and government test and quarantine strategy (Supplementary Fig. 8 and 9)37,45,46.
As the results could however provide complementary insights, we decided to add them to the current manuscript.

Randomization

Randamization was not applicable in this observational study. Participants were allocated to groups based on the described criteria.

Blinding

Blinding was not relevant to this observational study.

Reporting for specific materials, systems and methods

We require information from authors about some types of materials, experimental systems and methods used in many studies. Here, indicate whether each material, system or method listed is relevant to your study. If you are not sure if a list item applies to your research, read the appropriate section before selecting a response.

Materials & experimental systems

n/a	Involved in the study
☒	☐ Antibodies
☒	☐ Eukaryotic cell lines
☒	☐ Palaeontology and archaeology
☒	☐ Animals and other organisms
☐	☒ Clinical data
☒	☐ Dual use research of concern

Methods

n/a	Involved in the study
☒	☐ ChIP-seq
☒	☐ Flow cytometry
☒	☐ MRI-based neuroimaging

Clinical data

Policy information about clinical studies

All manuscripts should comply with the ICMJE guidelines for publication of clinical research and a completed CONSORT checklist must be included with all submissions.

Clinical trial registration	NA
Study protocol	NA
Data collection	NA
Outcomes	NA
